# Response of nitrogen-fixing water fern *Azolla* biofertilization to rice crop

**DOI:** 10.1007/s13205-014-0251-8

**Published:** 2014-09-26

**Authors:** K. Bhuvaneshwari, Pawan Kumar Singh

**Affiliations:** Center for Advanced Study in Botany, Banaras Hindu University (B.H.U.), Varanasi, 221005 India

**Keywords:** Rice, Water fern *Azolla*, Crop response, Soil enrichment

## Abstract

The water fern *Azolla* harbors nitrogen-fixing cyanobacterium *Anabaena azollae* as symbiont in its dorsal leaves and is known as potent N_2_ fixer. Present investigation was carried out to study the influence of fresh *Azolla* when used as basal incorporation in soil and as dual cropped with rice variety Mahsoori separately and together with and without chemical nitrogen fertilizer in pots kept under net house conditions. Results showed that use of *Azolla* as basal or dual or basal plus dual influenced the rice crop positively where use of fern as basal plus dual was superior and served the nitrogen requirement of rice. There was marked increase in plant height, number of effective tillers, dry mass and nitrogen content of rice plants with the use of *Azolla* and N-fertilizers alone and other combinations. The use of *Azolla* also increased organic matter and potassium contents of the soil.

## Introduction

The use of biofertilizers and green manures is not only a need for sustainable agriculture but also an environment friendly and economically feasible scientific method. The integrated use of organic and inorganic fertilizers is desirable to sustain crop yields and maintenance of soil health (Meelu and Singh 1991; Prasanna et al. 2008). *Azolla* is a free-floating water fern and has agronomic importance due to its ability to fix nitrogen (Singh 1977). It forms a nitrogen-fixing symbiosis with the cyanobacterium *Anabaena azollae*, which is present in the leaf cavity of the fern (Watanabe 1982, spore 1992). *Azolla* is grown as a green manure before transplantation of rice or as an intercrop with the rice. Both the practices are reported to increase the growth and yield of rice (Singh 1989; Singh and Singh 1995).

In Asia, *Azolla* is the most commonly used green manure for rice crop, due to its high growth rate, nitrogen-fixing capacity and ability to scavenge nutrients from soil and water. The fern doubles its biomass in 2–5 days under ideal environmental conditions (Watanabe et al. 1989). *Azolla* can supply more than half of the required nitrogen to the rice crop and besides providing nitrogen it is beneficial in wetland rice fields for bringing number of changes which include preventing rise in pH, reducing water temperature, curbing NH_3_ volatilization, suppressing weeds and mosquito proliferation (Pabby et al. 2004).

Therefore, the present study was undertaken to examine the feasibility of using *Azolla* as nutritional alternative for rice crop. Consequently, the main objective of this work was to study the influence of *Azolla* as green manure or dual crop on rice crop growth and development.

## Materials and methods

Pot incubation experiment was conducted between June and October 2012 under net house condition at the Institute of Agricultural Sciences, Banaras Hindu University (B.H.U.), Varanasi, India. Soil samples used in this experiment were collected from Khaira, a village near Varanasi, India. All the samples were brought to the laboratory, where sieving was done with 2-mm sieve. Part of the sieved soil was air dried and some chemical properties were determined (Table 1). Five kg of soil was weighed with a balance and kept into each, 48 pots having holes at the base. There were three pots per treatment and the control experiment was inclusive. Six seedlings of Mahsoori rice variety were transplanted in each pot. *Azolla* was used as green manure (basal) and dual (associated), at the rate of 0.012 kg pot^−1^ (2 ton ha^−1^) and in basal treatments it was incorporated in soil before transplanting. Equal shares of *A. pinnata* and *A. filiculoides* were mixed for achieving a more stable plant growth. A list of treatments along with the abbreviations used in the text and figure is given in Table 2.

**Table 1:** Initial soil properties at the start of the experiment

Soil parameters	Value
Organic matter (OM), %	3.12
Phosphorus (P), ppm	54.0
Calcium (Ca), kg ha^−1^	17.2
Potassium (K), kg ha^−1^	0.19
Magnesium (Mg), kg/ha^−1^	2.4
pH	6.6

**Table 2:** Details of treatments and abbreviations used in the text

S. no	Treatments	Abbreviations	Treatment no
1	Without nitrogen (Control)	0N	T1
2	30 kg ha^−1^ nitrogen	30N	T2
3	60 kg ha^−1^ nitrogen	60N	T3
4	90 kg ha^−1^ nitrogen	90N	T4
5	0N + Basal *Azolla* (BA)	0N + BA	T5
6	30N + Basal *Azolla* (BA)	30N + BA	T6
7	60N + Basal* Azolla* (BA)	60N + BA	T7
8	90N + Basal* Azolla* (BA)	90N + BA	T8
9	0N + BA + Dual* Azolla* (DA)	0N + BA + DA	T9
10	30N + BA + Dual* Azolla* (DA)	30N + BA + DA	T10
11	60N + BA + Dual* Azolla* (DA)	60N + BA + DA	T11
12	90N + BA + Dual* Azolla* (DA)	90N + BA + DA	T12
13	0N + Dual* Azolla* (DA)	0N + DA	T13
14	30N + Dual* Azolla* (DA)	0N + DA	T14
15	60N + Dual* Azolla* (DA)	0N + DA	T15
16	90N + Dual* Azolla* (DA)	0N + DA	T16

Nitrogen applied to each treatment:

0N: Control
30N: 0.18 g N pot^−1^ (30 kg ha^−1^)
60N: 0.36 g N pot^−1^ (60 kg ha^−1^)
90N: 0.54 g N pot^−1^ (90 kg ha^−1^)

The following measurements were performed:

- Plant height (at 100 % heading).
- Number of fertile tillers per plant (at 100 % heading).
- Dry mass.
- Nitrogen, phosphorus (P) and potassium (K) content.
- Soil chemical analysis (organic matter, pH, P, K, Ca and Mg).

Sampling was performed 45 days after incorporating *Azolla.*

The pH of the soil was determined using pH meter with glass-column combination electrode in distilled water and 0.01 M CaCl_2_ solution at a ratio of 1:2 soil solution. The organic matter was determined using Walkley and Black method (Jackson 1967). Total nitrogen was determined by Kjeldahl method (Jackson 1973). Exchangeable K, Ca and Mg were extracted using ammonium acetate, K was determined on flame photometer and Ca and Mg by EDTA titration.

Statistical analysis was performed by Tukey HSD test using the SPSS 16.0 (Statistical package for Social Sciences) software package.

## Results

**Fig. 1:**
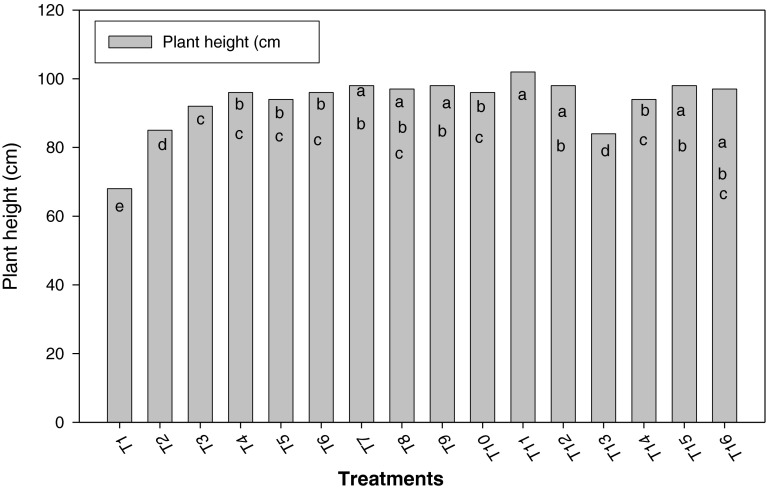
Influence of treatments on rice plant height

Figure 1 shows the behavior of the rice plant height, influenced by *Azolla* incorporation and/or association as well as different nitrogen (N) rates. An increase for this character is seen in treatments where incorporated and/or associated *Azolla* was used. However, nitrogen dose and *Azolla* influence did not show significant differences, expect for the treatments in which 30 kg ha^−1^ N doses with T_6_ incorporated *Azolla* and 60 kg ha^−1^ N doses with T_11_ incorporated and associated *Azolla* were applied. Treatments from T_7_ to T_12_ and T_14_ to T_16_, in which incorporated and/or associated *Azolla* was used, presented the greatest plant height values, without showing significant differences among them. Treatment T_9_ stood out, in which nitrogen was not applied and both ways of *Azolla* were performed. It was also observed that treatments where *Azolla* was used without nitrogen also showed better behavior with significant differences in relation to control, which presented a poor plant growth, just 67.25 cm high.

**Fig. 2:**
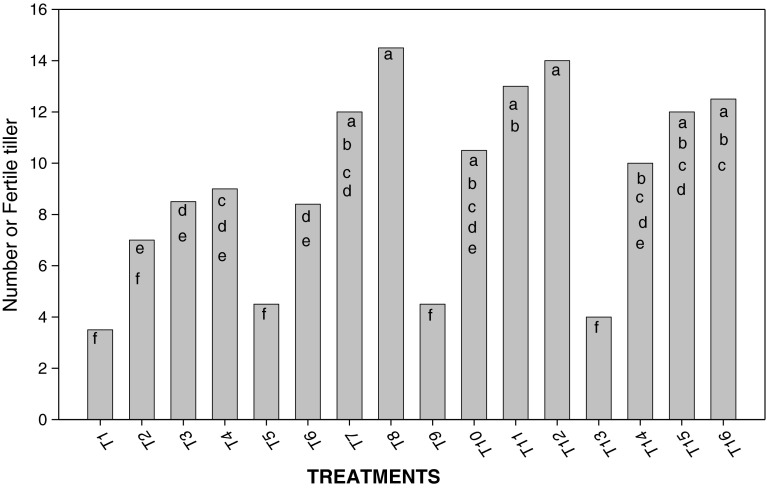
Influence of treatments on the number of fertile tillers *a*, *b*, *c*, *d*, *e*, *f*, *g*, *h*, *i* shows the significant differences on the basis of Tukey HSD

Figure 2 shows the number of effective tillers per plant. Nitrogen doses applied together with *Azolla* in this experiment influenced this variable as well. Treatments where *Azolla* was incorporated and/or associated (T_8_, T_12_, T_11_, T_16_, and T_7_) stood out, for presenting the highest number of tillers per plant. (All the data are significant *p* < 0.05).

**Fig. 3:**
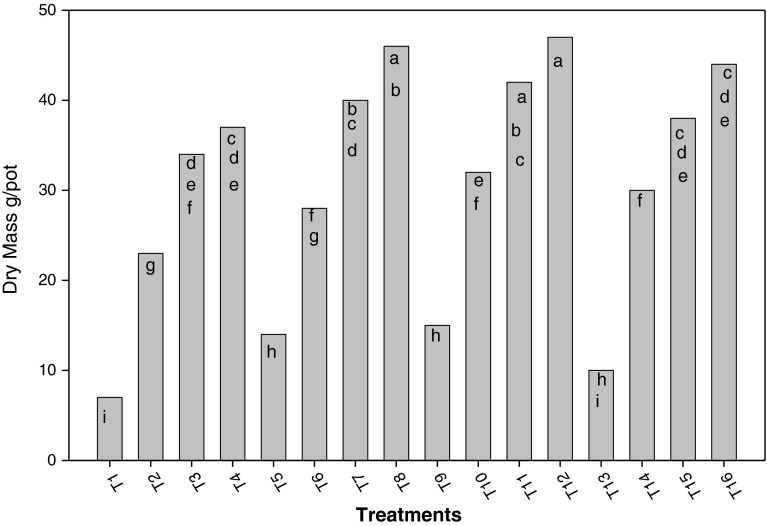
Influence of treatments on rice dry mass *a*, *b*, *c*, *d*, *e*, *f*, *g*, *h*, *i* shows the significant differences on the basis of Tukey HSD

**Fig. 4:**
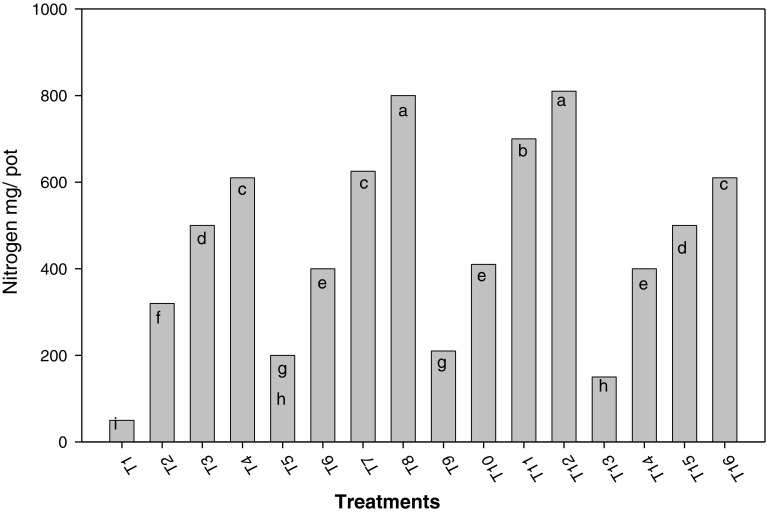
Influence of treatments on nitrogen content *a*, *b*, *c*, *d*, *e*, *f*, *g*, *h*, *i* shows the significant differences on the basis of Tukey HSD

Figures 3 and 4 show nitrogen extracted by rice plants and plant dry mass at different variants, up to 100 % flowering. Like the number of tillers per plant and height, nitrogenous fertilization along with *Azolla* influenced nitrogen extracted by plants and dry mass production. Treatments T_8_ and T_12_ presented the highest nitrogen concentrations in plants. In both of them, incorporated *Azolla* and incorporated and associated *Azolla* at 90 kg ha^−1^ N were used.

**Fig. 5:**
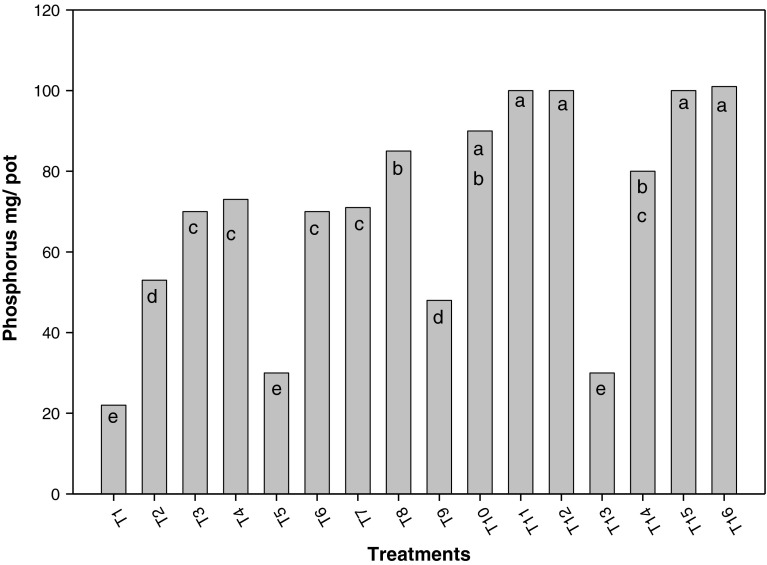
Influence of associated and incorporated Azolla on phosphorus contents in rice plants with different nitrogen doses *a*, *b*, *c*, *d*, *e*, *f*, *g*, *h*, *i* shows the significant differences on the basis of Tukey HSD

**Fig. 6:**
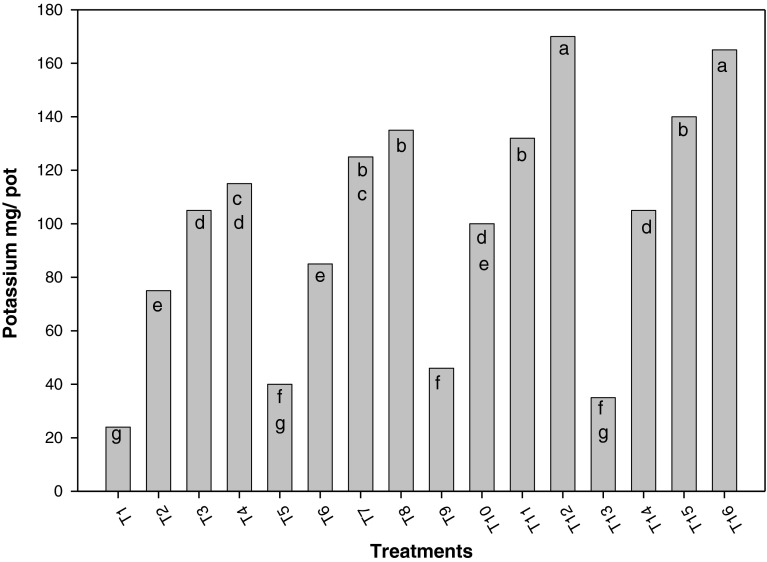
Influence of different treatments on potassium content in rice plants *a*, *b*, *c*, *d*, *e*, *f*, *g*, *h*, *i* shows the significant differences on the basis of Tukey HSD

Figures 5 and 6 show the influence of associated and/or incorporated *Azolla* on phosphorus and potassium contents in rice plants, with different nitrogen doses, where both nutrients presented similar behavior to that of nitrogen, positively influenced by N fertilization and *Azolla*. In general, combining both ways of using *Azolla* surpassed the remaining treatments, followed by variants where *Azolla* was associated to rice crop, and where fern was incorporated.

When analyzing soil chemical features after incorporating *Azolla* (Table 3), it could be noticed that treatments did not influence pH, P, Ca and Mg. However, they did influence organic matter and potassium content. In this sense, the highest values were achieved by combining incorporated and associated *Azolla*, followed by treatments where *Azolla* was incorporated, leaving the third place of treatments in which the fern was associated to rice crop.

**Table 3:** Soil characteristic features after harvesting

Treatments	pH	OM	P	K	Ca	Mg
T1	6.70c	3.91ghi	65.67abcd	0.23e	19.83	3.07
T2	5.72bc	3.89hi	64.33abcd	0.25cde	19.67	3.67
T3	6.72bc	3.77i	60.33cd	0.24e	20.33	3.67
T4	6.72bc	3.67i	66.67ab	0.25cde	19.33	3.75
T5	6.80ab	4.47cde	61.33bcd	0.34ab	19.50	3.00
T6	6.71bc	4.50cd	60.00cd	0.31ab	19.58	3.00
T7	6.78abc	4.85ab	61.00bcd	0.29bcd	19.67	3.04
T8	6.76abc	4.73bc	66.67ab	0.37a	19.50	3.00
T9	6.75abc	4.70bc	62.33bcd	0.35ab	20.08	3.00
T10	6.80ab	4.88ab	68.67a	0.31ab	19.50	3.10
T11	6.84a	5.12a	66.33abc	0.3b	19.50	3.10
T12	6.77abc	4.13fgh	62.33bcd	0.33ab	19.58	3.12
T13	6.82a	4.16fgh	64.33abcd	0.24de	19.33	3.04
T14	6.80ab	4.22def	61.67bcd	0.20e	19.58	3.58
T15	6.82a	4.28def	63.67abcd	0.30bc	19.25	3.54
T16	6.77abc	4.19efg	69.66a	0.31b	19.67	3.33

a, b, c, d, e, f, g, h, i shows the significant differences on the basis of Tukey HSD

## Discussion

An increase in plant height and number of tillers per plant was seen in the treatments where incorporated and/or associated *Azolla* was used. The highest value was achieved in treatment T_11_, where 60 kg ha^−1^ N was applied to rice plant (Figs. 1, 2). Treatment T_9_ stood out, in which nitrogen was not applied and both ways of using *Azolla* were performed. This treatment seems to supply the nitrogen demands of rice plants. In, general terms, such behavior of rice height was caused by the influence of nitrogen content in the medium, which could be supplied through different ways and means, as reported earlier (Singh 1989; Baker 1999; Bhuvaneshwari 2012; Bhuvaneshwari and Kumar 2013).

Like the number of tillers per plant and height, nitrogenous fertilization along with *Azolla* influenced nitrogen extracted by plants and dry mass production (Figs. 3, 4). Treatments combining both ways of using *Azolla* tended to produce more biomass than the remaining treatments with the same nitrogen dose. Therefore, combining incorporation and association of this fern not only serve to provide plants with significant amount of nitrogen, but also enables a better use of nitrogen added by mineral fertilization and thereby, a higher dry mass production. Similar results were recorded when *A. pinnata* was used by Manna and Singh (1990); Baker (2000); Bhuvaneshwari and Singh (2012).

For both above variables, it is highlighted the fact that nitrogen, phosphorus and potassium doses are inadequate for achieving good crop development, however, the influence of incorporated and/or associated *Azolla* allows a better use of nitrogen and better conditions for assimilating other nutrients, thus improving crop nutrition as reported by Samarajeewa (1999).

Treatment T_8_ and T_12_ presented the highest nitrogen concentration in the plants. In both of them, incorporated *Azolla* and 90 kg ha^−1^ N were applied. The incorporation of this fern seems to maintain a higher soil nitrogen availability than its association, as observed by other workers (Singh 1989; Fageria et al. 1999; Baker 2000).

Regarding dry mass production and nitrogen content, in treatments where incorporated and associated *Azolla* were combined, value tended to be higher, compared to other treatments presenting the same nitrogen doses. This is caused by the influence of incorporated and associated *Azolla* on plant available nitrogen content in the soil, as a result of nitrogen release during fern decomposition, reduction in loss of nitrogen might have occured  when applied as N fertilizer and that excreted into water by associated *Azolla* (de Macale et al. 2002).

Figures 5 and 6 show the influence of associated and incorporated *Azolla* on phosphorus and potassium contents in rice plants, combining both ways of using *Azolla* surpassed the remaining treatments, followed by the variants where *Azolla* was associated to rice crop, and where it was incorporated. This could be due to the influence of supplying these elements where fern is decomposed, as well as the effect on pH provoked by the associated *Azolla*, which increases the solubility of such elements. It was also reported that *Azolla* increased fertilizer efficiency, when applied in both ways (Pabby et al. 2004).

The soil chemical features are given in Table 3. Treatments did not influence pH, P, Ca and Mg. However, they did influence organic matter and potassium contents. Organic matter and potassium contents in the soil presented significant differences among treatments; the use of *Azolla* influenced both of them. The highest values were achieved by combining incorporated and associated *Azolla*, followed by treatments where *Azolla* was incorporated, third place was recorded given to the treatment in which fern was associated to rice crop. This is related to the contribution of *Azolla* after decomposition. Similar results were observed by other researchers (Singh 1979; Baker 1999).

The utilization of *Azolla* as a green manure benefits rice crop, obtaining the highest response when combining incorporation and association of this fern. It also favors nutrient absorption by rice and increases its yield, as well as organic matter content of the soil.
